# Fourteen- to Eighteen-Month-Old Infants Use Explicit Linguistic Information to Update an Agent’s False Belief

**DOI:** 10.3389/fpsyg.2019.02508

**Published:** 2019-11-20

**Authors:** Kyong-Sun Jin, Yoon Kim, Miri Song, Yu-Jin Kim, Hyuna Lee, Yoonha Lee, Minjung Cha, Hyun-Joo Song

**Affiliations:** ^1^Department of Psychology, Sungshin Women’s University, Seoul, South Korea; ^2^Department of Psychology, Yonsei University, Seoul, South Korea; ^3^Assesta Co., Ltd., Seoul, South Korea; ^4^Hugmom Psychology Consultation Institution, Seoul, South Korea

**Keywords:** infancy, false-belief understanding, theory of mind, verbal information, psychological reasoning, cognitive development

## Abstract

The current research examined how infants exploit linguistic information to update an agent’s false belief about an object’s location. Fourteen- to eighteen-month-old infants first watched a series of events involving two agents, a ball, and two containers (a box and a cup). Agent1 repeatedly acted on the ball and then put it in the box in the presence of agent2. Then agent1 disappeared from the scene and agent2 switched the ball’s location from the box to the cup. Upon agent1’s return, agent2 told her, “The ball is in the cup!” Agent1 then reached for either the cup (cup event) or the box (box event). The infants looked reliably longer if shown the box event as opposed to the cup event. However, when agent2 simply said, “The ball and the cup!” – which does not explicitly mention the ball’s new location – infants looked significantly longer if shown the cup event as opposed the box event. These findings thus provide new evidence for false-belief understanding in infancy and suggest that infants expect an agent’s false belief to be updated only by explicit verbal information.

## Introduction

Our psychological reasoning in everyday life is a dynamic process; we constantly update our own and others’ psychological states based on relevant information. In this updating process, much of the new information, especially the information about events or states that are not perceivable, is conveyed by communicative information such as the language we hear (Harris, 2012). Prior findings have shown that infants can use verbal information to update their own representations about unseen events (e.g., Ganea et al., 2007, 2016; Ganea and Harris, 2010, 2013). Infants can also update their representations about others’ psychological states such as goals based on others’ words (Song et al., 2014; Jin and Song, 2017).

Building on these previous findings, the present research investigated the types of linguistic information that infants consider as informative enough to update others’ false beliefs. In everyday life, our belief understanding is not a static snapshot of reality; we frequently update others’ beliefs when relevant information is provided through language. For instance, consider the classic false-belief, Sally-Anne task (Baron-Cohen et al., 1985). Sally hides a marble in a basket and then goes for a walk. While Sally is absent, Anne moves the marble out of the basket and puts it in a nearby box. Children’s false-belief understanding is assessed by the test question, “Where will Sally look for her marble when she returns?” The correct answer to this question is the basket, where Sally falsely believes the marble is. However, what if Anne kindly informed Sally of what she had done by stating, “Hey, I moved it! The marble is in the box!” As adults, we would readily expect Sally to update her belief and look for the marble in its current location. At what age do infants understand that an agent’s false belief can be updated by informative communication?

Accumulating evidence suggests that even infants may possess false-belief understanding (e.g., Onishi and Baillargeon, 2005; Southgate et al., 2007; Buttelmann et al., 2009; Kovács et al., 2010; Scott et al., 2010; Luo, 2011; Setoh et al., 2016). For instance, in Onishi and Baillargeon (2005), infants first saw an agent hide an object in box A as opposed to box B. Next, infants received a belief-induction trial in which the agent came to hold either a true or a false belief about the object’s location. In the test trial, the agent reached into either box A or box B. Infants expected the agent to reach into whichever box she *believed* the object was located, regardless of whether she held a true or a false belief. Positive evidence has now been obtained with children aged 6–25 months using a wide variety of response measures, leading many investigators to conclude that some ability to attribute false beliefs emerges early in life (for reviews, see Baillargeon et al., 2016; Scott and Baillargeon, 2017).

Less is known, however, about whether infants’ ability to understand others’ false beliefs is based on (1) rigid mechanisms of belief attribution (e.g., “seeing is believing”) or (2) more flexible mechanisms that allow attributed beliefs to be *updated* when relevant new information becomes available. To our knowledge, there has been only one study demonstrating that 18-month-old infants expect an agent’s false belief to be corrected by relevant verbal information (Song et al., 2008). In this study, the infants watched a series of events involving two agents (agent1, agent2), a ball, and two containers (a box and a cup). They first received three familiarization trials in which agent1 placed the ball inside the box as agent2 witnessed the scene. Next, in a belief-induction trial, agent1 was absent and agent2 moved the ball to the cup, which should have resulted in the attribution of a false belief about the ball’s location to agent1. The following intervention trial varied in two conditions. In the informative-intervention condition, agent2 told agent1 who had returned, “The ball is in the cup!” to communicate about the ball’s new location. In the uninformative-intervention condition, agent2 simply told agent1, “I like the cup!” During the final test trial which was identical in both conditions, agent2 disappeared from the scene and agent1 reached either for the box (box event) or the cup (cup event). In the informative-intervention condition, infants looked reliably longer at the box event than at the cup event; in the uninformative-intervention condition, the opposite pattern was found. Infants thus recognized that the utterance (“The ball is in the cup!”) in the informative-intervention condition was sufficient to update the agent’s false belief about the ball’s location. Although agent2 in the uninformative-intervention condition mentioned the cup (“I like the cup!”), the infants regarded this statement as including insufficient information about the ball’s new location. These results suggest that 18-month-old infants expect an agent’s false belief about an object’s location to be corrected when the agent receives a relevant communication about this location.

The primary goal of the present research was to better understand the nature of the linguistic information that allows infants to update others’ beliefs. In Song et al. (2008), it remains unclear how precise infants’ expectations are about what might constitute an informative communication. The utterance in the informative-intervention condition included the object (ball) and its location (cup) in the same sentence, while the utterance in the uninformative-intervention condition did not. This left open at least two possibilities. On the one hand, it might be that infants would consider any utterance mentioning both the ball and the cup (e.g., “The ball and the cup!”) as informative enough to update agent1’s false belief about the ball’s location. On the other hand, infants might expect agent1’s false belief to be updated only by a communication that explicitly states the ball’s new location (“The ball is in the cup!”). To explore these possibilities, we tested infants in two different conditions: a *complete-intervention condition* in which agent2 explicitly stated the ball’s new location, “The ball is in the cup!”, and an *incomplete-intervention condition* in which agent2 merely mentioned the ball and its location in a conjoined-noun phrase, “The ball and the cup!” We reasoned that the findings would further our understanding of the range of communicative information that infants can use to update others’ beliefs.

The secondary goal of the present research was to confirm the robustness of Song et al.’s (2008) findings. Given that Song et al. provided the first evidence that infants can update others’ false beliefs through linguistic information, it seems important to replicate their findings with a large sample and in a different language. The present research thus tested a relatively large sample (*N* = 30 per condition) of Korean-learning infants with a wide age range (14–18 months old), using a procedure similar to that used by Song et al. We reasoned that converging evidence across languages and ages would provide stronger support for infants’ ability to use language to update others’ beliefs and for infants’ false-belief understanding in general.

### The Current Research

**Figure 1:**
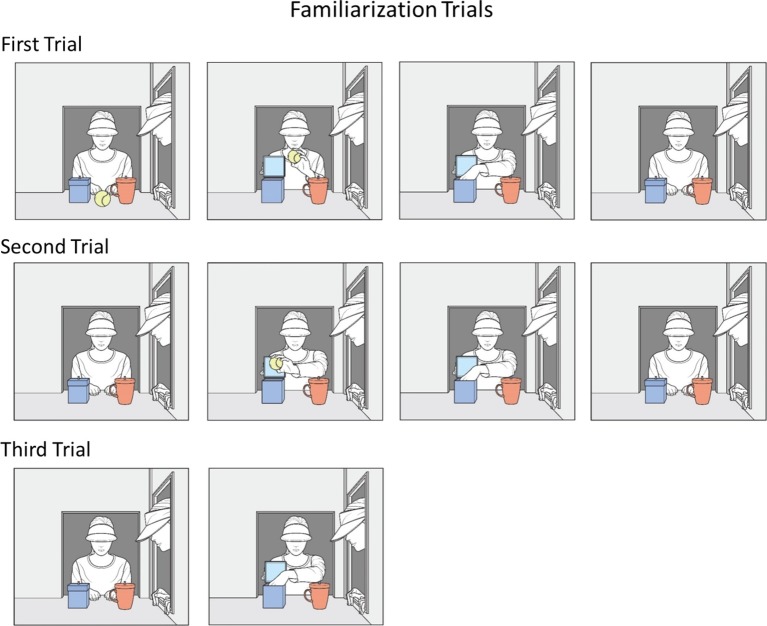
Schematic drawing of the events shown during the three familiarization trials.

**Figure 2:**
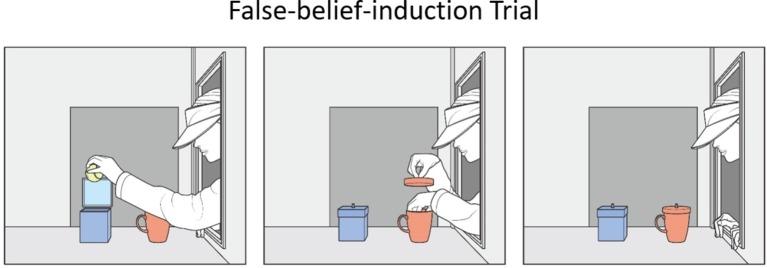
Schematic drawing of the events shown during the false-belief-induction trial.

**Figure 3:**
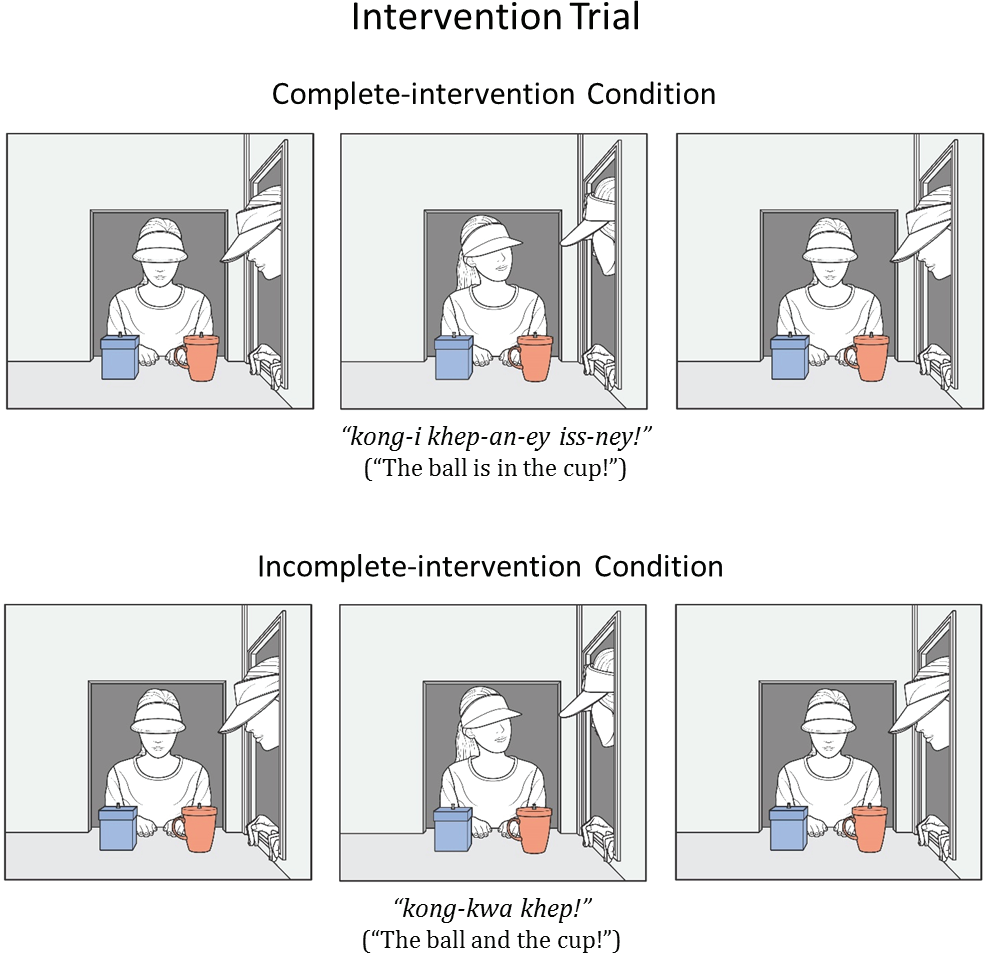
Schematic drawing of the events shown during the intervention trial in the complete-intervention and incomplete-intervention conditions.

In the current research, 14- to 18-month-old Korean infants were randomly assigned to a complete-intervention or an incomplete-intervention condition. The infants in both conditions first received familiarization trials in which a blue box and a red cup were seated on the apparatus floor. Agent1 placed a ball inside the box, while agent2 watched agent1’s action (see Figure 1). The infants next received a false-belief-induction trial in which agent1 was absent and the ball was moved to the cup by agent2 (see Figure 2). At this point, agent1 should falsely believe that the ball yet remained in the box. Next, the infants in the two conditions received a different intervention trial (see Figure 3). In the complete-intervention condition, agent2 told agent1 “kong-i khep-an-ey iss-ney!” (ball-SUBJ cup-interior-LOC exist-DECLAR; “The ball is in the cup!”), and she then repeated this same communication a second time. Notice that this communication could update agent1’s false belief about the object’s location. In the incomplete-intervention condition, agent2’s communication only mentioned the object and the location in a conjoined-noun phrase: specifically, agent2 simply told agent1 “kong-kwa khep” (ball-CONJ cup; “The ball and the cup!”) twice.

**Figure 4:**
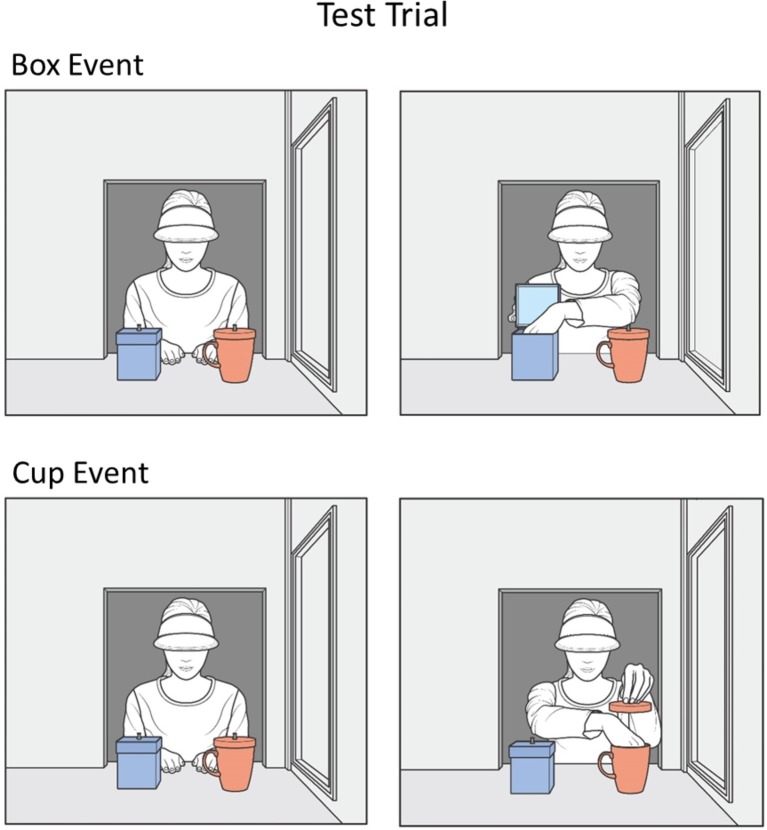
Schematic drawing of the events shown during the test trial.

Finally, the infants in both conditions received one test trial (see Figure 4) in which agent2 was absent and agent1 reached either for the box (box event) or for the cup (cup event). Our predictions were as follows. With respect to the complete-intervention condition, we expected that, as in Song et al. (2008), the infants would (1) realize that agent1 was absent during the false-belief-induction trial and hence possessed the false belief that it was still in the box and (2) understand that this false belief could be updated by agent2’s utterance (“The ball is in the cup!”) during the intervention trial. The infants should therefore expect agent1 to reach for the cup and detect a violation when she searched for the ball in the box instead. We thus predicted that the infants in the complete-intervention condition should look significantly longer if presented the box event in contrast to the cup event, as was found in Song et al. With respect to the incomplete-intervention condition, several possibilities existed. One was that the infants would view the simple mention of the object and its container (“The ball and the cup!”) as sufficient to correct agent1’s belief about the ball’s location, leading them to show the same looking pattern as in the complete-intervention condition. A second possibility was that the infants would completely ignore this incomplete-intervention utterance, expect agent1 to search for the ball in the box, where she wrongly believed the ball was still located, and hence look significantly longer at the cup than at the box event. Such a pattern would be similar to that found by Song et al. in their uninformative-intervention condition (“I like the cup!”). Finally, a third and intermediate possibility was that infants would view the incomplete-intervention utterance as ambiguous communication (e.g., it is insufficient to update agent1’s belief, even though it mentioned the object and its container); in this case, the infants might be unable to form a clear expectation about agent1’s actions, and they might then look equally at the two events.

## Methods

### Participants

Participants were 60 healthy term infants, 27 male and 33 female (*M* = 16 months, 27 days, range = 14 months, 0 day to 18 months, 29 days). Another 25 infants were excluded, because they were fussy (*n* = 9; four in the complete-intervention condition, five in the incomplete-intervention condition); inattentive (*n* = 11, eight in the complete-intervention condition, three in the incomplete-intervention condition); distracted (*n* = 1), because they had a test looking time that exceeded 3 *SD*s from the condition mean (*n* = 3; two in the complete-intervention condition and one in the incomplete-intervention condition); or because of parental interference (*n* = 1). Fifteen infants were randomly assigned to the four treatments formed by crossing the two conditions (complete-intervention or incomplete-intervention) and the two test events (box or cup) (complete-intervention condition, box event: *M* = 16 months, 27 days; range = 14 months, 17 days to 18 months, 24 days; complete-intervention condition, cup-event: *M* = 16 months, 27 days, range = 14 months, 18 days to 18 months, 29 days; incomplete-intervention condition, box event: *M* = 17 months, 0 day; range = 14 months, 0 day to 18 months, 24 days; incomplete-intervention condition, cup event: *M* = 16 months, 24 days; range = 14 months, 3 days to 18 months, 26 days).

Participants were recruited from the Seoul metropolitan area, Korea, *via* advertisements on online parenting communities or *via* flyers at public health centers. They were acquiring Korean as their native language. Parents were reimbursed for their travel costs. No compensation for participation was provided. The experiment was conducted in accordance with ethical guidelines and was approved by the institutional ethics review board at Yonsei University. Each infant’s parent signed informed consent prior to the test session.

### Apparatus

The apparatus consisted of a display booth (200 cm high × 95 cm wide × 64 cm deep) with a large opening (53 cm × 88 cm) in its front wall; between trials, a supervisor lowered a curtain in front of this opening. The sidewalls were covered with white muslin, the back wall was made of white foam board, and the floor was covered with beige adhesive paper.

Agent1 sat on a wooden chair centered behind a window (49 cm × 45.5 cm) in the back wall of the apparatus; a screen behind agent1 hid the testing room. The back window extended from the apparatus floor and was located 5 cm from the right wall. During the false-belief-induction trial, for which agent1 was absent, the back window was covered with a bright green panel; this was intended to help infants notice that agent1 was absent during the trial. Agent1 wore an orange shirt and a blue visor that hid her eyes from the infants.

Agent2 wore a blue shirt and an ivory visor and knelt on pillows behind a window (51 cm × 38 cm) in the right wall of the apparatus. This window was located 4 cm above the apparatus floor and 5.5 cm away from the back wall. During the test trial, the side window was covered with a muslin curtain.

The box and the cup were placed on the apparatus floor 15 cm in front of the back window and 7 cm from the back window’s left and right edge, respectively; the box and the cup were 10 cm apart from their closest points. The box (10 cm × 9 cm × 9 cm) was made of blue cardboard. Its lid was also made of cardboard and was 10 cm square and 2 cm thick. It was hinged to the back of the box with blue tape. The cup was made of red porcelain and the handle was facing left; it was 10 cm tall, 9 cm in diameter at the top, and 12.5 cm wide at its widest point (with its handle included). The cup’s lid was made of cardboard and was 1 cm tall and 9.5 cm in diameter; attached to the center of the lid was a small transparent knob (1 cm × 1 cm × 1 cm). A green tennis ball (6 cm in diameter) was placed 5 cm in front of the box and the cup and centered between both the objects.

We tested infants in a brightly lit room. During each session, one camera captured an image of the apparatus, while another captured an image of the infant. The images were checked by the supervisor to confirm that the correct events were displayed in each trial. The images were also recorded and checked offline for accuracy.

### Trials

All trials had an initial phase and a final phase. The infants’ looking times during the two phases were calculated separately. During the initial phase of a trial, the agents performed the prescribed actions and then paused; during the final phase, the infants watched the paused scene until the end of the trial. The duration of the initial phase was fixed based on the time it took to complete the specific actions. The duration of the final phase was infant-controlled (see below for the specific criteria used to end trials). When each trial ended, a supervisor lowered the curtain in front of the apparatus and stimuli were readied for the next trial.

In the following descriptions, the numbers in parentheses indicate the time (in seconds) it took to perform the described action. To help the agents comply with the script of the event, a metronome made a soft beat once per second.

#### Complete-Intervention Condition

##### First Familiarization Trial

At the beginning of the first familiarization trial, agent1 sat at the back window, and agent2 knelt at the window in the right wall. Agent1 faced forward, with her bare hands resting on the floor 9 cm behind and centered between the box and cup. Agent2 faced forward, with her left profile visible to the infants; her hands loosely grasped the lower edge of the window. Both agents looked at a neutral point between the box and cup. During the trial, agent1 gazed at the ball and box as she acted on them, while agent2 watched agent1’s actions; both agents wore visors and thus the infants could not see their eyes^1^.

The initial phase of the first familiarization trial lasted 15 s. After a pause (1 s), agent1 grasped the ball (1 s) with her left hand, lifted it to a position about 20 cm above the apparatus floor between the two containers (1 s), and tilted it from left to right twice, changing orientation once per second (4 s). Agent1 then grasped the box’s lid (1 s) with her right hand and opened the box (1 s). Next, agent1 placed the ball into the box (2 s) with her left hand and then returned her left hand to the apparatus floor (1 s). Finally, agent1 closed the box’s lid (2 s) with her right hand and returned her right hand to the apparatus floor (1 s). The two agents then paused in their respective positions. During the final phase of the trial, the infants watched this paused scene until the trial ended.

##### Second Familiarization Trial

The initial set-up of the second familiarization trial was identical to that of the first trial, except that the ball was placed inside the box. The initial phase again lasted 15 s. After a pause (1 s), agent1’s right hand grasped the box’s lid (1 s) and opened it (1 s). Agent1’s left hand then reached into the box (1 s), pulled out the ball and lifted it above the box (1 s), and tilted it from left to right twice (4 s). Next, agent1 lowered the ball back inside the box (2 s) and withdrew her left hand to the apparatus floor (1 s). Finally, agent1 closed the box’s lid (2 s), and withdrew her right hand to the apparatus floor (1 s). The two agents then paused, and the infants watched this paused scene until the trial ended.

##### Third Familiarization Trial

The initial set-up of the third familiarization trial was identical to that of the second trial. The initial phase lasted 3 s. To start, agent1’s right hand grasped the box’s lid (1 s) and opened it (1 s). Next, her left hand reached into the box (1 s). The two agents then paused, and the infants watched this paused scene until the trial ended.

##### False-Belief-Induction Trial

The set-up at the start of the false-belief-induction trial was identical to that of the third familiarization trial, except that agent1 was now absent and the back window was covered by the green panel. The initial phase lasted 12 s. Agent2’s right hand grasped the box’s lid (1 s) and opened it (1 s). She then reached inside the box with her left hand (1 s), pulled out the ball, and brought it toward herself, to a position about 19 cm above the apparatus floor, 9 cm from the cup, and 15 cm from the side window (1 s). Next, agent2 closed the box (1 s) with her right hand, grasped the cup’s lid (1 s), and lifted it about 9 cm above the cup (1 s). Using her left hand, she then placed the ball inside the cup (2 s), and returned her left hand to its starting position at the window ledge (1 s). Finally, agent2 replaced the cup’s lid with her right hand (1 s) and returned her right hand to the bottom edge of the side window (1 s). Agent2 then paused, and the infants watched this paused scene until the trial ended.

##### Intervention Trial

The set-up at the start of the intervention trial was identical to that of the second and third familiarization trials. The intervention trial only had an initial phase, which lasted 12 s. After a pause (1 s), the two agents looked at each other (1 s). Agent2 who was a female native speaker of Korean then said “공이 컵 안에 있네!” (*kong-i khep-an-ey iss-ney*; ball-SUBJ cup-interior-LOC exist-DECLAR; “The ball is in the cup!”) (3 s) twice, with a pause (1 s) after each utterance. She spoke in an infant-directed manner at a comfortable listening level of about 68 dB (measured with a sound-level meter placed at the infant’s location, before each experiment session). Next, both agents turned their heads back to look at the neutral position between the two containers (1 s) and paused (1 s), and then the trial ended.

There was no final phase in the intervention trial because a paused scene could suggest that agent1 had lost her interest in the ball or had failed to understand agent2’s utterance.

##### Test Trial

The initial set-up of the test trial was similar to that of the intervention trial except that only agent1 was present; agent2’s window was covered with a muslin curtain. During the trial whose initial phase lasted 3 s, the infants saw either a box or a cup event. In the box event, using her right hand, agent1 grasped the box’s lid (1 s) and opened it (1 s); with her left hand, she then reached inside the box (1 s). Agent1 then paused, holding the lid with her right hand while her left hand was still inside the box. The infants watched this static scene until the end of the trial. In the cup event, agent1 grasped the cup’s lid with her left hand (1 s) and lifted it up about 9 cm (1 s); she then reached inside the cup with her right hand (1 s) and then paused until the end of the trial.

#### Incomplete-Intervention Condition

The infants in the incomplete-intervention condition received the same trials as those in the complete-intervention condition, with one exception: During the intervention trial, agent2 said “공과 컵!” (*kong-kwa khep*; ball-CONJ cup; “The ball and the cup!”) (2 s) twice, resulting in a 10-s initial phase.

### Procedure

During the experiment, each infant sat on his or her parent’s lap in front of the apparatus; the infant’s head was approximately 50 cm from the apparatus. Parents were instructed to close their eyes and to remain silent and neutral during the entire experiment.

Each infant’s looking behavior was monitored by two naïve observers hidden behind large frames on either side of the apparatus; the primary observer’s responses were used to determine the ending of the trials. Looking times during the initial and final phases of each trial were calculated separately.

All infants first received the three familiarization trials described above. Examination of the infants’ looking times during the initial phase of each trial revealed that they were highly attentive: they looked on average for 14.59/15 s during the first trial, 14.47/15 s during the second trial, and 2.59/3 s during the third trial. The final phase of each trial ended when the infant (1) looked away for 2 consecutive seconds after having looked for at least 2 cumulative seconds, or (2) looked for 60 cumulative seconds without looking away for 2 consecutive seconds.

Next, all infants received the false-belief-induction trial described above. They were very attentive during the initial phase and looked for 11.64/12 s on average. The final phase ended when the infant (1) looked away for 2 consecutive seconds after having looked for at least 3 cumulative seconds, or (2) looked for 60 cumulative seconds without looking away for 2 consecutive seconds.

Following the false-belief-induction trial, the infants received the intervention trial appropriate for their condition. As before, the infants were highly attentive during the initial phase and looked for 11.43/12 s in the complete-intervention condition and 9.39/10 s in the incomplete-intervention condition (as mentioned before, this trial had no final phase).

Finally, each infant received one test trial; half of all the infants in each condition saw the box event, and half saw the cup event. During the initial phase, they looked for 2.55/3 s on average, suggesting that they were quite attentive. The final phase ended when the infant (1) looked away for 2 consecutive seconds after having looked for at least 2 cumulative seconds, or (2) looked for 40 cumulative seconds without looking away for 2 consecutive seconds.

To assess interobserver agreement during the familiarization and test trials, the final phase of each trial was divided into 100-ms intervals, and the computer determined whether the two observers agreed if the infant was or was not looking at the event for each interval. The percentage of agreement was calculated for each trial by dividing the number of intervals in which the observers agreed by the total number of intervals in the trial. The percentage of agreement was measured for 55 infants (only one observer was available for the other five infants^2^) and averaged 93% per trial per infant.

Preliminary analyses of the test data revealed no main effect of infants’ sex or whether infants’ age was above or below the median and no interactions involving the two factors, all *F*s(1, 52) < 2.78, *p*s > 0.10; the data were therefore collapsed across the two factors in subsequent analyses.

## Results

The infants’ looking times during the final phases of the three familiarization trials were averaged and analyzed by means of a 2 × 2 analysis of variance (ANOVA) with condition (complete- or incomplete-intervention) and test event (box or cup) as between-subject factors. No effect was significant, all *F*s(1, 56) < 2.84, *p*s > 0.09, suggesting that the infants in the four experimental groups tended to look equally during these trials (complete-intervention/box event, *M* = 28.95, *SD* = 13.58; complete-intervention/cup event, *M* = 25.62, *SD* = 12.54; incomplete-intervention/box event, *M* = 22.05, *SD* = 12.30; incomplete-intervention/cup event, *M* = 29.56, *SD* = 11.36). A similar analysis of the infants’ looking times during the final phase of the false-belief-induction trial also revealed no significant effects, all *F*s(1, 56) < 2.07, *p*s > 0.15, suggesting that they looked about equally during this trial (complete-intervention/box event, *M* = 17.79, *SD* = 14.12; complete-intervention/cup event, *M* = 13.55, *SD* = 7.61; incomplete-intervention/box event, *M* = 11.12, *SD* = 6.78; incomplete-intervention/cup event, *M* = 14.39, *SD* = 10.36).

**Figure 5:**
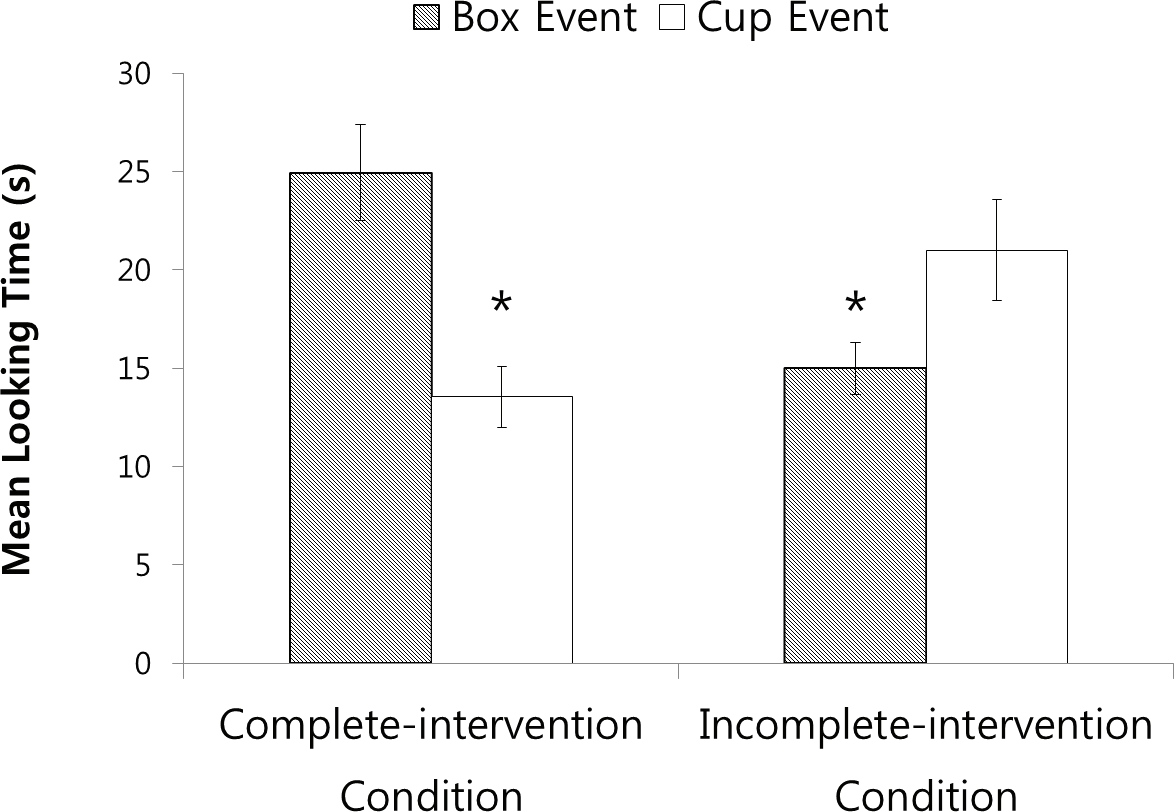
Infants’ mean looking times for the box and cup test events in the complete- and incomplete-intervention conditions. Error bars represent standard errors.

The infants’ looking times during the final phase of the test trial (see Figure 5) were analyzed as mentioned above. Neither the main effect of condition nor that of event was significant, *F*s(1, 56) < 1.73, *p*s > 0.19. However, the Condition × Event interaction was significant, *F*(1, 56) = 18.06, *p* < 0.001, $\eta_{p}^{2}$ = 0.24. Planned comparisons indicated that in the complete-intervention condition, the infants who saw the box event (*M* = 24.94, *SD* = 9.49) looked reliably longer than those who saw the cup event (*M* = 13.54, *SD* = 6.01), *F*(1, 56) = 15.47, *p* < 0.001, *d* = 1.44. In the incomplete-intervention condition, the infants who saw the cup event (*M* = 21.00, *SD* = 10.02) looked reliably longer than those who saw the box event (*M* = 14.98, *SD* = 5.04), *F*(1, 56) = 4.31, *p* < 0.05, *d* = 0.76. Non-parametric Wilcoxon rank-sum tests confirmed the results of the complete-intervention (*Z* = 3.13, *p* < 0.01) and incomplete-intervention (*Z* = 1.68, *p* < 0.05, one-tailed) conditions.

Finally, an analysis of covariance (ANCOVA) showed the same test results, a significant Condition × Event interaction, after adjusting for the differences in the infants’ looking times during the three familiarization trials, *F*(1, 55) = 14.44, *p* = 0.00, $\eta_{p}^{2}$ = 0.21, or the false-belief-induction trial, *F*(1, 55) = 15.16, *p* = 0.00, $\eta_{p}^{2}$ = 0.22.

In sum, in the complete-intervention condition, infants expected agent1’s false belief about the ball’s location to be updated when she was informed of the ball’s location in the sentence, “The ball is in the cup!”, confirming the results of Song et al. (2008). In contrast, in the incomplete-intervention condition, infants expected that agent1 would not look for the ball in the correct location (cup) when she was simply told the object and the location in the phrase, “The ball and the cup!”, suggesting that the infants assumed the phrase was not sufficient to update agent1’s false belief. This result in the incomplete-intervention condition also rules out low-level interpretations of the result in the complete-intervention condition (e.g., infants looked longer at the box event because they preferred the box to the cup).

## General Discussion

Infants in the present experiments demonstrated their ability to use verbal information to update an agent’s false belief. In many previous experiments on infants’ understanding of others’ false belief (Onishi and Baillargeon, 2005; Buttelmann et al., 2009; Kovács et al., 2010; Surian and Geraci, 2012), infants expected an agent to hold a true belief only when he or she witnessed a specific event (e.g., a change in an object’s location). For example, in the true-belief conditions of Onishi and Baillargeon (2005), the agent watched a watermelon move from one place to the other. However, people can often learn about the world even when they do not directly experience things. Newly received linguistic information is one of the most common sources of information that people take into account when they update their representation about the world. The present findings added to prior evidence (Song et al., 2008) that infants expect an agent’s false belief to be updated when the agent is provided with relevant verbal information without observing the referred event.

Consistent with the current findings, several experiments have shown that infants can update their own representations about physical or psychological states through language (e.g., Ganea et al., 2007, 2016; Ganea and Harris, 2010, 2013; Song et al., 2014; Jin and Song, 2017). For example, Ganea et al. (2016) showed that infants as young as 16 months can update their representation of a visual presentation based on a verbal description of a change in that presentation (e.g., “Now the dog goes to the table! The dog is on the table!”). In Jin and Song (2017), 12-month-olds updated their representations of others’ goals by using others’ words: they expected an actor to change her goal object in her upcoming actions if she changes her word from one to another before performing goal-oriented actions. The present findings suggest that infants use linguistic information not only to update their own mental representations but also to expect such updates in others.

The critical question of the present research was what infants view as informative linguistic communication that can update others’ beliefs. To address this question, we chose to compare the two similar, complete-intervention (“The ball is in the cup!”) and incomplete-intervention (“The ball and the cup!”) utterances. The two utterances contained exactly the same content words (ball, cup) just in different structures. Nonetheless, the infants interpreted only the complete-intervention utterance, but not the incomplete-intervention utterance, as informative enough to update the agent’s false belief. The results provide information concerning the nature of the information infants extract from language. They were able to differentiate the sentence (“The ball is in the cup!”) from the conjoined-noun phrase (“The ball and the cup!”) and concluded that only the sentence was informative communication. The phrase, despite mentioning the ball and the cup, was taken as insufficient to inform agent1 about the ball’s new location. The results suggest that infants form a specific representation of the utterance meaning rather than a rough one when hearing the utterances. These findings are consistent with previous research showing that infants can use detailed linguistic information to determine the events described by utterances. During the second year of life, infants use word order (e.g., Hirsh-Pasek and Golinkoff, 1996), function morphemes such as determiners (Gerken and McIntosh, 1993; Kedar et al., 2006), or spatial prepositions (Meints et al., 2002) when finding a visual scene that matches spoken utterances they hear. The current research suggests that Korean-acquiring infants use their knowledge about sentence structures and function words to develop accurate representations of utterance meaning.

Given the current controversies surrounding various false-belief findings (e.g., Baillargeon et al., 2018; Poulin-Dubois et al., 2018), it was necessary to replicate and extend the evidence that infants can update false beliefs. Consistent with Song et al. (2008), the infants in the present research expected that agent1’s false belief about the ball’s location could be updated by an explicit utterance (“The ball is in the cup!”) from agent2. When agent2 simply mentioned “The ball and the cup!”, the infants expected agent1 to maintain her false-belief about the ball’s location. It is worth noting that many of the recent failures to replicate infants’ false-belief understanding involved anticipatory-looking tasks (e.g., Dörrenberg et al., 2018; Grosse Wiesmann et al., 2018; Kulke et al., 2018). In case of the failed replications in the violation-of-expectation tasks (Poulin-Dubois et al., 2013; Yott and Poulin-Dubois, 2016; Dörrenberg et al., 2018; Powell et al., 2018), a closer look into the details of the experiments suggests that some failed replications have tended to be caused by inadvertent procedural changes that could influence the ease of processing events (see Baillargeon et al., 2018 for a recent debate on this issue).

The current research suggests that infants’ false-belief understanding is based on flexible mechanisms that allow attributed beliefs to be updated when new relevant information becomes available. These findings provide support for the accounts that early false-belief understanding is flexible, context-sensitive, and readily integrated with information available from other cognitive processes such as memory and language comprehension (e.g., Scott et al., in press). These one-system accounts assume that our psychological reasoning is guided by a single mentalistic system that emerges early in infancy (e.g., Baillargeon et al., 2015; Carruthers, 2016, 2018; Scott and Baillargeon, 2017). According to such accounts, infants’ false-belief understanding mostly observed in spontaneous-response tasks is qualitatively similar to that of older children or adults observed in more traditional tasks. For example, an expanded processing-demands view (e.g., Setoh et al., 2016) explains that young children fail at traditional tasks because these tasks pose additional processing demands, such as response-generation and inhibitory control. Consistent with this view, improvements in various executive function skills including working memory and inhibitory control contribute to children’s success in traditional tasks (Carlson and Moses, 2001; Devine and Hughes, 2014; Duh et al., 2016). Infants’ success in the current violation-of-expectation task may also rely on working memory or executive function capacities. Infants must be able to abstract, store, and update an agent’s belief as situational information changes over trials. Such infants’ updating ability may be related to the refreshing capacities, one of the important executive functions. For instance, infants in the current experiments had to refresh and foreground the updated beliefs when reasoning about the agent’s test action (e.g., Raye et al., 2007). A potentially fruitful line of future research would be to examine how executive function or working memory influences false-belief reasoning in infancy.

For adults, the conjoined-noun phrase (“The ball and the cup!”) used in the incomplete-intervention condition could be interpreted as indirect communication about the ball’s location. In future research, we plan to examine the range of indirect communication that infants would interpret as informative in updating others’ beliefs. Would older infants take the phrase as an indirect hint that is relevant for the agent’s search? Or would infants need better indirect statements or communicative settings to update attributed beliefs? In case of nonverbal communication, 18-month-old infants used an agent’s indirect requests (e.g., the agent ostensively shows an infant a key that they both knew could be used to open a locked box) to understand her actual goal (e.g., to ask the infant to take the key and open the box to retrieve the toy inside) (Schulze and Tomasello, 2015). Interestingly, infants were less likely to interpret the agent’s accidental acts (e.g., the agent accidentally pushes the key in front of an infant) as such indirect communication. These results suggest that infants can interpret an agent’s acts as indirect communication only when her acts appropriately display her communicative intentions. Future research should examine at what age and under what pragmatic circumstances infants can make inferences from various types of verbal information.

To illustrate, we can examine whether infants use sentences including novel content words to update others’ beliefs. Previous experiments have shown that infants assume that conversational partners follow communication principles, for example, that conversational partners should be trustful, informative, relevant, and clear (Grice, 1975), and interpret the utterances with the novel words as intending to communicate the object’s location (Martin et al., 2012; Vouloumanos et al., 2014; Pitts et al., 2015). In Pitts et al. (2015), for example, 20-month-old infants expected a speaker’s sentence with a novel word for a location (e.g., “The ball is in the *blicket*!”) would convey information about the object’s location to a listener who had no knowledge about the object’s location. In future research, we will examine how infants interpret sentences comprising unknown words (e.g., “The ball *acorp* the box!”; “The ball is in the *dax*!”; or “The *mido acorp* the *dax*!”) in the present scenario.

Our findings have some implications for the generalizability of false belief understanding in infants. Note that our Korean 14- to 18-month-old infants showed the same results as American 18-month-olds in Song et al. (2008). So, we obtained the same pattern of results as in Song et al. (2008) despite variations in participants’ age and ethnicity. One may wonder whether Korean-speaking infants might display earlier abilities to use linguistic information in updating others’ false beliefs than English-speaking infants due to some linguistic variations. Currently available data, however, do not permit us to draw a conclusion on whether this may be the case. Both Korean- and English-acquiring infants begin to produce spatial terms in their language at similar ages (Fenson et al., 1994; Pae and Kwak, 2011) and such similar developmental trajectory can lead to a prediction that English-speaking 14- to 18-month-olds should display similar patterns to the Korean infants in the present research. Or, it is also possible that some cross-linguistic differences in location terms can affect children’s abilities to use linguistic information in false-belief understanding over the course of development. There are syntactic/semantic distinctions made with Korean locative case markers, which Korean children must learn through exposure to their language. For instance, Korean post-nominal locative markers distinguish the location of static verbs (e.g., −ey for being in a room) from motion verb location (e.g., −eyse for playing in a room), while both of these events are described by the same word in in English (for other examples, see Bowerman and Choi, 2001). In addition, there is a strong verb bias for the locative marker “-ey”: it typically occurs with static verbs such as “issta.” Therefore, Korean children must learn these distinctions when acquiring locative markers. Future research can examine whether such language-specific learning would lead to cross-linguistic differences in the development of abilities to exploit linguistic information in updating others’ beliefs.

In sum, the present research suggests that infants use detailed representation of linguistic information to update an agent’s false belief about an object’s location. Infants expected an agent’s false belief to be updated when the agent was informed through a direct sentence that made explicit the object’s location, but not through a phrase that simply mentioned the object and the location in the same utterance. These findings suggest that soon after their first birthdays, young infants are able to use relevant communicative information to reason about others’ beliefs.

## Data Availability Statement

The datasets generated for this study are available on request to the corresponding author.

## Ethics Statement

The studies involving human participants were reviewed and approved by the institutional ethics review board at Yonsei University. Written informed consent to participate in this study was provided by the participants’ legal guardian/next of kin.

## Author Contributions

H-JS developed the study concept and design. Data collection was performed by K-SJ, YK, MS, Y-JK, HL, YL, and MC. Data analysis was performed by K-SJ, who also drafted a first manuscript. H-JS, K-SJ, and HL provided critical revisions. All authors approved the final version of the manuscript for submission.

### Conflict of Interest

MS was employed by company Assesta Co., Ltd. and YK was employed by company Hugmom Psychology Consultation Institution.

The remaining authors declare that the research was conducted in the absence of any commercial or financial relationships that could be construed as a potential conflict of interest.

The reviewer YL declared a past co-authorship with one of the authors H-JS.

## Footnotes

^1^As our goal was to investigate the effect of verbal information on infants’ false-belief understanding, the experimenters wore visors to control the influence of other social cues such as eye gaze.

^2^Two offline observers coded the infants’ test looking times from the videos to confirm the accuracy of the primary observer’s measurement.
